# Innovation, advertising, personal selling, and sustainability in the industries massively benefited from major public health emergencies: paradoxical evidence from China

**DOI:** 10.3389/fpubh.2023.1186026

**Published:** 2023-10-05

**Authors:** Guangying Xie, Cancan Zhang, Qianqian Fang, Xiaole Tang, Yani Zhang

**Affiliations:** ^1^School of Economics and Management, China University of Mining and Technology, Xuzhou, China; ^2^School of Business, Dongguan City University, Dongguan, China; ^3^School of Finance, Zhengzhou College of Finance and Economics, Zhengzhou, China; ^4^Chaobo Medical Materials Farming Cooperatives of Yangcheng, Jincheng, China

**Keywords:** innovation, environmental sustainability, social sustainability, governance sustainability, advertising, personal selling, profitability, COVID-19 pandemic

## Abstract

**Background:**

While major public health emergencies have severe socio-economic impacts, they may also present many opportunities for certain industries. For these industries that have benefited significantly (e.g., China’ s healthcare industry), the traditional emphasis on improving business performance through increased investment in innovation, marketing and sustainability may face contextual applicability challenges.

**Methods:**

We used the data of healthcare industry in China during Covid-19 and the methods of hierarchical regression, moderating effect test to analyze the impact of innovation, advertising, personal selling, and sustainability on healthcare firms’ profitability. Three kinds of robust test including increasing the measurement range of variables, changing the data source and parameter estimation method, and Quantile regression are used.

**Results:**

This paper finds that innovation, advertising, and environmental sustainability have significant negative impacts on profitability, while personal selling, social sustainability, and governance sustainability have significant positive impacts on profitability in the industries massively benefited from major public health emergencies. Besides, social sustainability can significantly moderate the relationship between innovation and profitability.

**Conclusion:**

On one hand, for companies in industries that have benefited greatly from major public health emergencies, a shift in resource allocation from innovation, advertising, and environmental sustainability to personal selling, social sustainability, and governance sustainability may be more conducive to improving their profitability. On the other hand, for public health regulatory authorities, it is necessary to strengthen the supervision of sales representatives of health care enterprises, hospitals, public health organizations, etc., and appropriately subsidize the innovation of enterprises to enhance their innovation motivation.

## 1. Introduction

The new crown pneumonia epidemic has lasted for more than 3 years, and has had a serious impact on the social economy, business operations and residents’ lives. Academically, most of the existing literature is focused on how to survive from the dilemma, while neglects the research on the small number of industries and organizations that have gained significant market opportunities during this crisis. For the industries that have profited greatly from this major public health emergency, they are not only facing a massive increase in market demand, but also being in a scenario where most sectors of society are in recession or distress. This is a special situation in which “flowers wither away, and only this side of the scenery is good” in the process of economic crisis, which is significantly different from the rapid increase in market demand during the economic boom.

Resource allocation and optimization is a classic research topic in enterprise operation management and strategic decision-making (1–3). With limited resources, companies always need to reallocate resources according to the market environment to achieve optimal performance (4, 5). Innovation and marketing (including advertising and personal selling) are two traditional aspects that enterprises need to invest resources in. In general, both innovation and marketing investments can contribute to the profitability of a business. However, in an extremely favorable and extremely unfavorable market environment, the innovation and marketing behavior of enterprises may be largely affected as there may be more opportunism and survival pressure. If a company does not respond correctly to the changes in the external environment, it is likely to adversely affect its profitability. When the economy, society and most industries are suffering from major public health emergencies, it is interesting to study how to make decisions about the investment of innovation and marketing resources for industries that have benefited greatly. In particular, if these companies continue to invest more in innovation and marketing, will they be able to get more return on investment in this scenario? This is the main research gap that this paper fills in, which is seldomly studied in the past. On the other hand, the public has increasingly attached more importance to sustainability in recent years (6). Moreover, studies have shown that sustainability performs better in alleviating a company’s financial crisis in times of economic crisis than the regular situations (7). Therefore, it is necessary and meaningful to take sustainability into consideration when analyzing the effects of resource allocation on profitability, which may complement the research on sustainability strategies in the context of major public health emergencies from the perspective of beneficiary industries and companies.

Healthcare companies, as providers of healthcare products and services, are also important members of the public health system. Benefiting from the industry opportunities brought by the new crown pneumonia epidemic, China’s healthcare companies have achieved significant growth in net profit, return on assets, stock price and other financial performance indicators. Studying their innovation and marketing resource allocation and sustainable development behavior in this context can not only enrich the theory of organizational management and resource allocation of healthcare enterprises, but also have positive enlightenment for industry supervision and macro-control in the public health sector.

By using the data of China’s healthcare industry, this paper mainly has the following findings and contributions. First, this study finds that for the industries massively benefited from major public health emergencies, innovation investment will have a negative impact on the profitability of current period. However, conventional wisdom is that innovation can improve many corporate financial performance indicators, including profitability (8, 9). This deviation, which is contrary to the traditional conclusion, provides a new contextual perspective for dialectical innovation investment decisions. Moreover, the paper furtherly finds that this adverse impact is negatively moderated by social sustainability. Second, for the two marketing tools including advertising and personal selling, this research finds that in the context of major public health emergency, advertising has a negative effect on profitability in the benefited industries. This also contradicts the conclusion that advertising investment can promote corporate performance proposed by most previous studies in general situations (10, 11), and provides a new contextual factor for a scientific view of the impact of advertising on corporate performance. Third, this paper confirms that the positive impact of personal selling, social sustainability, and governance sustainability, as well as the negative impact of environmental sustainability on corporate profitability remains applicable in industries that benefit greatly from major public health emergencies. We believe these findings can make a positive contribution to filling research gaps in innovation, marketing, and sustainability decision-making in industries that benefit from major public health emergencies, and to enriching the specific context of innovation, marketing, and sustainability research more broadly.

The rest of this paper is organized as follows: Section 2 presents a systematic literature review against the impact of innovation, advertising, personal selling, and sustainability on profitability and proposes corresponding hypotheses. Section 3 illustrates the data collection process, variables measurement, and research methodology. Section 4 shows the statistical results and Section 5 discusses the findings. Section 6 explores the insights, and concludes this article with a discussion on research limitations and future directions.

## 2. Literature review and hypotheses development

Business operations is a process of converting resource inputs into outputs and making profits from it. For enterprises, the stragegy of resources allocation is so important that it may directly decide their output performance. This paper mainly discusses the allocation strategy of three types of key resources investment and their impacts on profitability: innovation, marketing, and sustainability. Among them, marketing investment mainly focuses on advertising and personal selling. In the following, this part will review the relevant research in these four aspects in turn, and put forward research hypotheses and the theoretical model based on the situational characteristics of industries that benefit largely from major public health emergencies.

### 2.1. The impact of innovation on profitability in the industries that massively benefited from major public health emergencies

Innovation is a concept often mentioned in economics and management, which contains organizational innovation, market innovation, process innovation, product innovation, and service innovation, and plays an important role in promoting economic development and social progress (12, 13). Academically, the existing literature has fully studied the relationship between innovation and enterprise performance in general contexts and find that, under most circumstances, innovation, including incremental and fundamental innovation, can promote the improvement of business performance (14, 15). Specifically, for the profitability, which reflects the ability of an enterprise to obtain returns by using its resources, has also been found by many literatures in recent years that it is significantly affected by innovation (16, 17).

However, some scholars hold the opposite perspective towards the relationship between innovation and performance. For example, Link argues that in view of fierce market competition, especially the imitation of competitors, if companies spend high sums of money to innovate, it can sometimes have a negative impact on the future development of the company (18). Kessler holds a similar view, arguing that the impact of enterprise innovation investment on enterprise performance may be affected by external factors such as the company’s infrastructure, scale, and industry attributes. Only when external factors are favorable can enterprises obtain more profits in the market through innovative investment (19). In addition to market competition and external environmental factors, the ability of enterprises to use technology is also an important factor. Generally, enterprise innovation investment does not have a direct impact on performance, but needs to be transformed. Only enterprises with strong technology application ability can promote innovation investment in a direction conducive to improving corporate performance (20). Besides, Xu’s et al. study also found that the impact of innovation on financial performance is not significant in large enterprises, and it will show a negative impact in small enterprises (21).

In addition, there are also some studies showing that the relationship between firms’ innovation input and their financial performance is nonlinear or insignificant. For instance, Lee et al. (22) and Yoo et al. (23) found that the relationship between firm’ s R&D (Research & Development) expenditure and its ROA (Return on Asset) performance is nonlinear, and influenced by home region orientation and firm lifecycle stages. Koellinger found that despite there is indeed a certain correlation between innovation input, business revenue increase and employment rate improvement, if the enterprise operating revenue is used as the measurement, innovation investment does not necessarily bring more profits to the enterprise, and there is no significant positive correlation between it and enterprise performance (24).

Therefore, as innovation is often long-term and being slow in the process from resource input to output benefit, it may not always lead to better financial performance in the short term. Particularly, if profitability is used as a measuring indicator, the company’s R&D revenue may not be able to compensate for R&D costs, resulting in a negative correlation between the two. This negative correlation may become more pronounced in the industries that hugely benefited from suddenly arisen market opportunities, for example, the healthcare industry during COVID-19. This is because if companies ignore market opportunities and continue to focus intensively on R&D, even if they do not consider the risk that the epidemic will end after the development of products related to the treatment of the new crown pneumonia epidemic, there is still the risk that competitors will crowd out market share and reduce innovation revenues and profitability. Thus, we propose the following hypotheses,

*Hypothesis 1*: In the industries that massively benefited from public health emergencies, innovation would have a negative impact on profitability during the emergency.

### 2.2. The impact of advertising on profitability in the industries that massively benefited from major public health emergencies

Advertisements are messages paid for by those who send them and are intended to inform or influence people who receive them, as defined by the Advertising Association of the UK (25). From this definition, we can clearly see that advertising costs money, and for enterprises, it requires resource investment. However, in most cases, this investment is worth it and can lead to better financial performance for the business. In recent years, Cici-Karaboga and Sekeroglu found that advertising expense spending has a significant boost to financial performance based on the data of well-known listed companies (10). Mirza et al. also found that not only the current advertising expenditure has a significant role in promoting the financial performance of enterprises, but also the advertising investment in the past period would still play a residual role (26). Niu and Ma’s research found that advertising expenditures can promote the positive effects of technology on financial performance, playing a significant moderating role between the two (27).

Nevertheless, some studies have also found that the impact of advertising on corporate performance is not significant or negative. For example, Pourkarimi and Kam found that increasing advertising expenditures alone had no significant effect on market share and sales performance (28). Based on the data from Indian pharmaceutical industry, Pal and Nandy’s research also indicate that the impact of advertising on ROA performance is not significant (29). Besides, Xu’s et al. research found that for small companies, advertising expenditure can put economic pressure on the business, which may have a negative impact on performance (21). Moreover, the effectiveness of corporate advertising is also affected by advertising content and efficiency (30, 31).

In major public health emergencies, on the one hand, consumers tend to actively increase their search for related products and services due to concerns about their own safety, thereby reducing the marginal utility of the advertising promotion function. On the other hand, major public health events often lead to economic recession, business closures and lower income levels in the affected regions. For enterprises that have benefited from the crisis, if they do not actively assume social responsibility but to advertise their products and services in large quantities, they may easily attract consumer disgust. Therefore, we propose the following hypothesis,

*Hypothesis 2*: In the industries that massively benefited from public health emergencies, advertising would have a negative impact on profitability during the emergency.

### 2.3. The impact of personal selling on profitability in the industries that massively benefited from major public health emergencies

In marketing practice, companies often employ multiple marketing methods at the same time, including advertising, personal selling, sales promotion, public relations (32, 33), etc. Among them, personal selling is the use of sales agents to personally deliver messages to target audiences. Despite the complex marketing techniques and online sales, salespeople still play an important role in building relationship with customers and expressing personal care, attention, emotional contact, etc., which are essential in marketing (34, 35). The current literature has amply demonstrated that personal selling has a positive effect on reversing negative public perceptions, promoting product information, and ultimately promoting sales and improving business performance (36–38).

Compared to advertising, personal selling is better targeted and private. In major public health emergencies, beneficiary enterprises can better compensate for the deficiencies of advertising and promote sales through targeted door-to-door marketing. Moreover, during major public health events, consumers often go out less frequently to reduce the risk of infection. Taking the initiative to sell products to the customer’s address can also better meet the needs of customers, especially some personalized needs, thereby enhancing the profitability of enterprises. Consequently, we propose the following hypothesis,

*Hypothesis 3*: In the industries that massively benefited from public health emergencies, personal selling would have a positive impact on profitability during the emergency.

### 2.4. The impact of sustainability on profitability in the industries that massively benefited from major public health emergencies

With the advancement of industrialization and the attention to environmental and social issues, sustainable development has increasingly become a common goal pursued by countries around the world, and the concept of sustainability has gradually been accepted by all sectors of society. At present, the mainstream view in academia is that sustainability mainly includes three dimensions: environmental sustainability, social sustainability, and governance sustainability (39, 40). In the firm level, sustainability is connected to the extent to which enterprises and their products and service quality meet the environmental, social and governance dimensions, and it has been closely related to the impacts that an enterprise can provide to the society when playing a state role and substituting the function of government from the macro perspective (41, 42). Most existing studies on the relationship between sustainability and corporate performance reveal a positive correlation. For example, Chelawat et al. found that good sustainability performance of enterprises is positively correlated with their financial performance (43). Xie et al. also believe that there is no doubt about the positive correlation between environmental, social and governance sustainability activities and a company’s asset returns performance (44). De Lucia et al. found that there is a positive correlation between sustainability practices and financial indicators such as ROA and ROE (Return of Equities) (45). Recently, Zhang et al. found that the enterprises with good sustainability performance will gain a relative competitive advantage during financing, and can obtain funds at a lower financial cost, saving the operating cost of the enterprise, which is conducive to improving the performance of the enterprise (46). Zhang et al. discovered that sustainability may enhance firm performance by alleviating financial constraints, improving external financing, and sending high quality signals to the credit market (47). When the major public health emergencies occur, companies that hugely benefited can also improve their reputation, corporate image, and boost sales and increase profitability by performing with high ratings on sustainability. Hence, we propose the following hypothesis,

*Hypothesis 4*: In the industries that massively benefited from public health emergencies, sustainability would have a positive impact on profitability during the emergency.

In fact, corporate sustainability not only directly improves profitability, but also influences the relationship between innovation, advertising, personal selling, and profitability. Innovation plays positive role in the long-term development of enterprises, industries and even the economy and society, and is one of the important ways for the sustainable development of enterprises. Companies that emphasize sustainability tend to have a long-term layout and higher requirements for innovation, which is less likely to be significantly reduced by short-term input–output efficiency. During major public health emergencies, companies in industries facing large market opportunities are likely to be detrimental to short-term market competition and financial performance if they continue to focus on innovation and ignore the market. This is more evident in highly sustainable companies. Hence, we propose the following research hypothesis:

*Hypothesis 5*: In the industries that massively benefited from public health emergencies, sustainability would negatively moderate the relationship of innovation and profitability during the emergency.

Companies with good sustainability not only have positive impact on financial performance, but also usually have the characteristics of good credit quality and strong anti-risk ability (48). Extant research manifests that sustainability would contribute to customer satisfaction, corporate image, and reputation. In the process of marketing, customer cognition and satisfaction of the enterprise may also have an important impact on marketing effect and product sales. Therefore, sustainability may play a positive role in the relationship between advertising, personal selling, and profitability. In the context of major public health emergency, most businesses and residents are facing greater economic pressure. As a company benefiting from major public health emergency, it is necessary to establish a good corporate image to avoid the negative perception that this company is making a fortune during the disaster in the eyes of the public. A good sustainability rating can also promote the consumer recognition of a company and purchasing behavior. Hence, we propose the following research hypotheses:

*Hypothesis 6*: In the industries that massively benefited from public health emergencies, sustainability would positively moderate the relationship of advertising and profitability during the emergency.

*Hypothesis 7*: In the industries that massively benefited from public health emergencies, sustainability would positively moderate the relationship of personal selling and profitability during the emergency.

**Figure 1:**
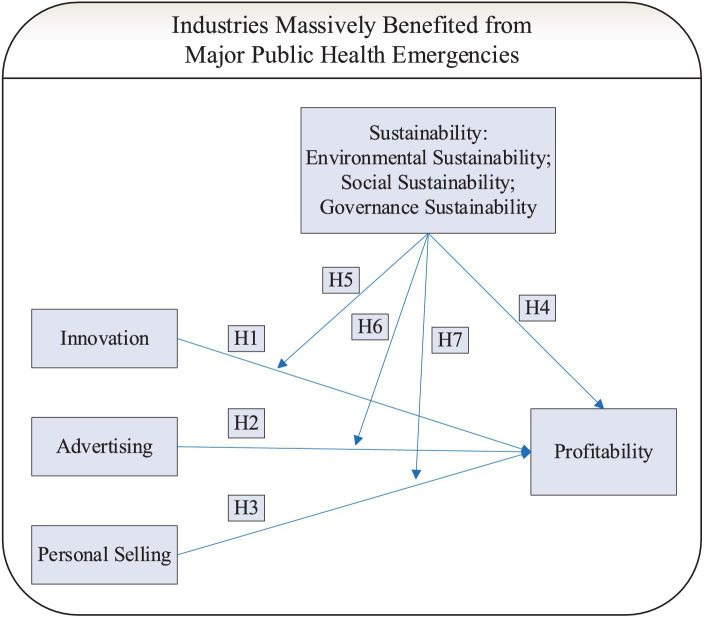
The research model.

Many studies have also found that firm age, firm size, and financial leverage have a significant impact on the firm’s performance (49, 50), but whether this effect is positive or negative is influenced by many factors and the environment (51–53). Specifically, studies often come to the opposite conclusion when it comes to profitability. In the studies on the age of firms, Ismail et al. found that as companies age, they have more efficient manufacturing capabilities and higher return on investment (54), but Loderer’s et al. research found that the profitability of a business declines with age (55). In the studies about firm size, on one hand, Kuncová et al. (56), Ibhagui & Olokoyo (57) and Mubeen et al. (58) found that large firms outperformed small firms in profitability, and firm size can positively moderate the relationship between factors such as financial leverage, product market competition and firm investment performance. On the other hand, Dhawan (59) and Fan’s et al. (60) studies found that firm size is negatively correlated with company performance. Similarly, there may also be a positive or negative relationship between financial leverage and profitability. Fosu (61) and Iqbal & Javed’s (62) studies found that financial leverage’ s impacts on profitability is positive, while Shahzad et al. (63) and Mathur’s et al. (64) studies obtained the opposite results. Considering that these factors may also have significant impacts on profitability, we included them as control variables in the empirical model for analysis in this study. Finally, the research model of this study is shown in Figure 1.

## 3. Materials and methods

### 3.1. Samples and data collection

We calculated the specific ROA data and their changes of listed companies in various industries of China’s A-share market between 2019 (the year before the outbreak) and 2021 (the second year after the outbreak of the new crown epidemic, latest data) first to find the industries that massively benefited from COVID-19. Through the statistics of a total of 4,929 companies across 11 industries, we find that the healthcare industry ranks first with an ROA growth rate of 68.86%, the materials industry ranks second with an ROA growth rate of 14.96%, and the average growth rate of 11 industries is −5.46%. Consequently, the healthcare industry is the only one that has gained high growth during the pandemic, and we conducted empirical research on this industry.

Healthcare industry is an aggregation and integration of sectors within the economic system that provides goods and services to treat patients with curative, preventive, rehabilitative, and palliative care, which includes the generation and commercialization of goods and services for maintaining and re-establishing health (65). In China, the healthcare industry is mainly made up of two main categories of companies. One category is healthcare equipment and service enterprises, including healthcare equipment, healthcare supplies, healthcare providers and services, healthcare technology, etc. The other category is pharmaceutical, biotechnology and bioscience enterprises, including traditional Chinese medicine, modern medicine, biotechnology, bioscience tools and services, etc. We used the data of China healthcare industry in this study. According to Wind database, as of December 31, 2021, there were 484 listed companies. Wind is a comprehensive database company of China, whose data are widely used in academic researches (66–68). Finally, after excluding samples containing missing values, 364 companies were selected. From the average level, the ROA of 364 companies selected by this study is 9.22%, which is basically equal to the mean value of 9.02% in the whole healthcare industry, indicating that the sample of this study has a good representativeness. All data used in this article is secondary data from publicly available database and can be openly accessed and verified.

### 3.2. Variables

The dependent variable in this study is profitability, which reflects the ability of a firm to generate revenue from resources. In financial analysis, there are many indicators to measure profitability, including return on assets, return on net assets, operating profit margin, cost and expense margin, surplus cash protection multiple, etc. Among them, return on assets is the most widely used variable, which reflects the ability of enterprises to use all assets to obtain profits. Compared with net profit, operating income, sales profit margin and other indicators, ROA can more intuitively reflect the input–output efficiency of enterprise resources.

In this study, we mainly analyzed four explanatory variables. The first is innovation. Most of the empirical studies draw on the practices of Miller and Le Breton-Miller (69) and measure the ratio of annual R&D expenditures to annual sales. Using this approach, we measured innovation with the ratio of R&D expenditure to sales revenue in 2021. We normalized the variable using the following formula:

Normalized Innovation*_i_* = (Innovation*_i_*-mean(innovation))/(Max (Innovation)-Min (Innovation)) (*i* = 1,2, 3…).

The second explanatory variable is advertising. For listed companies, advertising is an important tool in corporate marketing, and its related expenses are usually clearly listed in the financial report. Referring to the calculation method of innovation, we calculate advertising using the ratio of advertising cost to product sales revenue. During data analysis, this variable is also normalized so that its value ranges from 0–1.

The third explanatory variable is personal selling. Generally, the expenditure of enterprises on personal selling has a significant positive correlation with the proportion of salesmen. Considering that personal selling expenditure data is not directly disclosed in the company’s annual report, we use the ratio between the number of sales personnel and the total number of employees as a proxy variable. Likewise, this variable is normalized during data analysis so that its value ranges from 0–1.

The fourth explanatory variable in this study is sustainability. In general, there are two main methods for measuring sustainability in the existing literature. One is to directly quote the assessment values of third-party institutions, which is becoming more reliable and recognized as sustainability becomes more important in society and the maturity of assessment methods, data collection scope, etc. (70, 71). Another method is to design empirical indicators by the researchers themselves according to the definition of sustainability. By strictly following the dimensions and content of the concept and collecting data through questionnaire and other methods, it can cover small and medium-sized enterprises and non-listed companies that are rarely involved in third-party institutions (12, 72). Since the samples in this study are all publicly listed companies, we took the first measurement method, which directly references Wind’s sustainability rating data. Specifically, based on the division of Wind, we divide sustainability into three dimensions: environmental sustainability, social sustainability, and governance sustainability.

Considering that a firm’s age, size, and risk-taking can also have an impact on its profitability, we included them in our study as control variables (51–53). Among them, firm age is measured by the number of days from the date of firm registration to January 1, 2021, and we performed logarithmic processing on the original data. In terms of firm size, we control two main variables: asset value and number of employees. The corresponding data comes directly from the company’s annual report and is logarithmic processed before analysis. The last control variable is debt to net worth ratio, which measures the debt risk afford by shareholders and is calculated as the ratio between total liabilities and shareholders’ equity. The descriptive statistics of the variables are shown in Table 1.

**Table 1:** The descriptive statistics of variables.

Variables	1	2	3	4	5	5.1	5.2	5.3	6	7	8	9
1. Profitability	1.00	–	–	–	–	–	–	–				
2. Innovation	−0.34^***^	1.00	–	–	–	–	–	–				
3. Advertising	−0.18^***^	0.03	1.00	–	–	–	–	–				
4. Personal Selling	0.09	0.01	0.18^***^	1.00								
5. Sustainability	0.21^***^	0.02	−0.14^***^	0.01	1.00	–	–	–				
5.1 Environmental Sustainability	−0.03	−0.08	0.05	−0.06	0.65^***^	1.00						
5.2 Social Sustainability	0.23^***^	0.08	−0.14^***^	0.03	0.85^***^	0.36^***^	1.00					
5.3 Governance Sustainability	0.21^***^	0.04	−0.14^***^	0.04	0.68^***^	0.23^***^	0.36^***^	1.00				
6. Ln (Firm Age)	−0.10^**^	−0.20^***^	0.01	0.01	−0.01	0.12^**^	−0.10^*^	−0.01	1.00			
7. Ln (Asset Size)	0.08	−0.10^*^	−0.16^***^	0.05	0.25^***^	0.39^***^	0.08	0.20^***^	0.20^***^	1.00		
8. Ln (Total Number of Employees)	0.05	−0.10^*^	−0.17^***^	0.09^*^	0.25^***^	0.40^***^	0.08	0.16^***^	0.26^***^	0.84^***^	1.00	
9. Debt to Net Worth Ratio	−0.10^*^	−0.20^***^	−0.004	0.08	−0.10^*^	0.049	−0.12^**^	−0.06	0.15^***^	0.05	0.08	1.00
Mean	9.02	0.02	0.15	0.29	0.42	0.24	0.44	0.66	8.89	12.87	7.47	68.16
Minimum	−55.31	0	0	0	0	0	0	0	6.82	10.46	4.91	201.08
Maximum	113.06	1	1	1	1	1	1	1	9.68	16.61	10.76	−708.22
Std. Deviation	13.35	0.06	0.20	0.21	0.16	0.22	0.21	0.13	0.34	1.05	1.06	3298.76
Valid N	364	364	364	364	364	364	364	364	364	364	364	364

***p < 0.01, **p < 0.05, and *p < 0.10, two tailed test.

### 3.3. Research methodology

This study mainly focuses on the impact of innovation, advertising, personal selling, and sustainability on firm profitability in the industries that massively benefited from major public healthcare emergencies. We first conducted statistics on the changes in the return on assets of various industries in China’s A-shares since the outbreak of the new crown pneumonia epidemic, and found that the healthcare industry is one of the three industries that have achieved growth in 11 major industries, and the growth rate is far higher than that of other industries. Therefore, we use the healthcare industry as sample source and hierarchical regression (ordinary least squares regression), moderating effect test and robust test as statistical methods. Based on the theoretical review and research hypotheses, the empirical analysis of this study mainly includes three parts: (1) to test the impact of innovation, advertising, personal selling and sustainability on corporate profitability; (2) to test the moderating effect of sustainability and its sub-items on the relationship between innovation, advertising, personal selling and profitability; (3) Robustness tests are carried out by increasing the measurement range of sustainability variable, changing data source and parameter estimation method, as well as quantile regression.

## 4. Results

### 4.1. The impact of innovation, advertising, personal selling, and sustainability on profitability

We used the ordinary least squares estimation method and hierarchical regression models to analyze the impact of innovation, advertising, personal selling and sustainability, and Table 2 shows the detail regression results. Specifically, Model 1 is a model with only control variables, and Model 2 is the model containing control variables, innovation, advertising, personal selling, and sustainability; Model 3 breaks down sustainability of Model 2 into environmental sustainability, social sustainability, and governance sustainability.

**Table 2:** OLS and hierarchical regression results.

Dependent variable: Profitability	Model 1	Model 2	Model 3
Constant	27.908	58.008^***^	29.922
Independent Variables:			
Innovation		−86.603^***^	−91.676^***^
Advertising		−11.567^***^	−7.837^**^
Personal Selling		8.357^***^	5.906^**^
Sustainability		14.513^***^	
Environmental Sustainability			−10.840^***^
Social Sustainability			14.247^***^
Governance Sustainability			14.453^***^
Control Variables:			
Ln (Firm Age)	−4.307^**^	−5.849^***^	−5.164^***^
Ln (Asset Size)	1.799	1.047	1.273
Ln (Total Number of Employees)	−0.454	−1.164	−0.337
Debt to Net Worth Ratio	−0.006	−0.010^***^	−0.010^***^
Model-fitting metrics:			
*R* ^2^	0.030	0.2451	0.2902
Adjusted *R*^2^	0.019	0.2281	0.2701
*F* value	2.75**	14.41***	14.43^***^
Observations	364	364	364

***p < 0.01, **p < 0.05 two tailed test.

It can be seen from Model 2 that, on one hand, innovation and advertising investment have a significant negative impact on profitability, which proves the H1 and H2. On the other hand, personal selling and sustainability play a significant positive role in profitability, which verifies the correctness of H3 and H4. Model 3 once again confirms the negative impact of innovation and advertising on profitability, and the positive impact of personal selling. For the specific three dimensions of sustainability, Model 3 manifests that environmental sustainability has a negative impact on profitability, while social sustainability and governance sustainability have a positive impact on profitability. These results conflict with the positive impact of innovation and advertising on firm performance in the general context, but validates the hypotheses H1 and H2. For the four control variables, firm age and debt to net worth ratio are proved to have negative impact on profitability according to the Table 2, which is consistent with the studies of Shahzad et al. (63), Loderer et al. (55), Mathur et al. (64) and so forth. This suggests that both older and more risky companies perform relatively poorer in terms of profitability. Besides, the two variables measuring firm size including asset size and employee quantity are found insignificant to profitability.

### 4.2. The moderating effect of sustainability

In Section 4.1, we confirmed the role of sustainability in boosting corporate profitability. As a matter of fact, sustainability may also moderate the relationship between innovation, advertising personal selling and profitability. In this section, we analyze whether there are moderating effects by incorporating the interaction of sustainability with innovation, advertising, and personal selling into the regression models. Particularly, Model 4 is the regression of profitability on innovation, advertising, personal selling, sustainability, the product of sustainability and innovation, the product of sustainability and advertising, the product of sustainability and personal selling, and four control variables. Model 5 builds on Model 4 and further analyzes the moderating effect of sustainability on innovation, distinguishing between environmental sustainability, social sustainability, and governance sustainability. Results are shown in Table 3.

**Table 3:** Sustainability and its sub-items’ moderating effects.

Dependent variable: Profitability		Coef.	Std. Err.	*t*	*P* > |*t*|
Model 4 (*R*^2^ = 0.268, *F* = 11.72, Sig < 0.001, *N* = 364)	Constant	60.771^***^	18.223	3.33	0.001
	Innovation	99.416^*^	58.089	1.71	0.088
	Advertising	−11.302	10.182	−1.11	0.268
	Personal Selling	12.403^*^	7.499	1.65	0.099
	Sustainability	27.032^***^	7.438	3.63	0.000
	Sustainability *Innovation	−537.538^***^	165.363	−3.25	0.001
	Sustainability *Advertising	−1.359	24.974	−0.05	0.957
	Sustainability *Personal Selling	−8.400	17.266	−0.49	0.627
	Ln (Firm Age)	−5.876^***^	1.897	−3.10	0.002
	Ln (Asset Size)	1.128	1.095	1.03	0.304
	Ln (Total Number of Employees)	−1.542	1.117	−1.38	0.168
	Debt to Net Worth Ratio	−0.009^***^	0.003	−2.92	0.004
Model 5 (*R*^2^ = 0.312, *F* = 12.18, Sig < 0.001, *N* = 364)	Constant	28.541	18.547	1.54	0.125
	Innovation	126.008	150.135	0.84	0.402
	Advertising	−8.059^**^	3.275	−2.46	0.014
	Personal Selling	6.616^**^	2.908	2.27	0.024
	Environmental Sustainability	−7.481^*^	3.945	−1.90	0.059
	Social Sustainability	19.480^***^	3.976	4.90	0.000
	Governance Sustainability	16.852^***^	5.864	2.87	0.004
	Environmental Sustainability*Innovation	−215.977	144.415	−1.50	0.136
	Social Sustainability*Innovation	−281.360^**^	141.499	−1.99	0.048
	Governance Sustainability*Innovation	−120.217	224.370	−0.54	0.592
	Ln (Firm Age)	−5.382^***^	1.883	−2.86	0.005
	Ln (Asset Size)	1.413	1.078	1.31	0.191
	Ln (Total Number of Employees)	−0.677	1.097	−0.62	0.538
	Debt to Net Worth Ratio	−0.009^***^	0.003	−2.88	0.004

***p < 0.01, **p < 0.05, and *p < 0.10, two tailed test.

**Figure 2:**
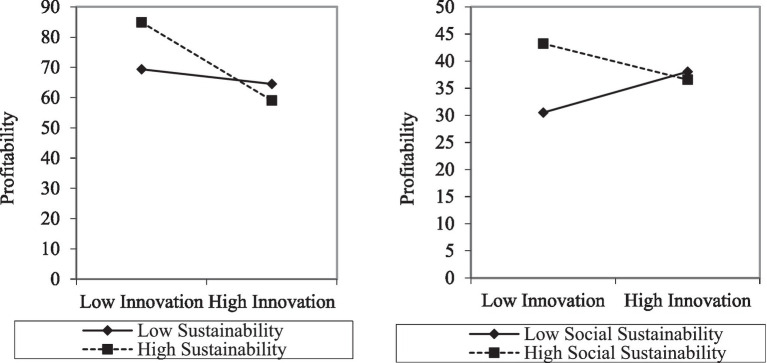
The moderating effect of overall sustainability and social sustainability.

In model 4, when the interaction items of sustainability and innovation, advertising and personal selling are added, only the coefficient of the interaction item of innovation and sustainability is significantly less than 0, and the regression coefficient of the interaction items of advertising and promotion and sustainability are not significant. This indicates that sustainability has a negative moderating effect on the relationship between innovation and profitability, but it does not significantly moderate the relationship between advertising, personal selling, and profitability. Therefore, the H5 is proved to be right while H6 and H7 are not supported by our data. Furthermore, Model 5 specifically demonstrates the moderating effect of the three dimensions of sustainability on the relationship between innovation and profitability. Judging from the statistical results, the moderating effect of environmental sustainability and governance sustainability on the relationship between innovation and profitability is not significant, while social sustainability has a significant negative moderating effect. That is, for companies with high social sustainability, their profitability will gradually diminish as investment in innovation increases. But for companies with low social sustainability, increased levels of innovation can still boost profitability. This indicates that the negative moderating effect of sustainability on the relationship between innovation and profitability is mainly caused by the dimension of social sustainability, as can be seen in the Figure 2.

Finally, we collate the validation result of the 7 hypotheses presented in Section 2, as shown in the Table 4.

**Table 4:** Hypotheses test result.

Hypothesis	Supported or Not supported
H1	YES
H2	YES
H3	YES
H4	YES
H5	YES
H6	NO
H7	NO

### 4.3. Robustness check

#### 4.3.1. Increasing the measurement range of sustainability

In the regression analysis above, we use internationally widely used concept of sustainability to analyze its impact on profitability. This indicator mainly includes three dimensions of environmental, social and governance factors. In recent years, public opinion, regulatory penalties, and legal proceedings have attracted more and more attention from consumers and the external public, and have significantly affected corporate image and business performance. Therefore, Wind added the dimension of dispute events to the traditional three dimensions of sustainability, and calculated the comprehensive score of sustainability. We use the comprehensive sustainability score calculated from these four dimensions to replace the traditional sustainability score, and then observe the impact of each related factor on profitability. Results are shown in Table 5.

**Table 5:** Robust test results by increasing the measurement range of sustainability.

Dependent variable: Profitability	Model 6	Model 7
Constant	48.793^***^	44.630^**^
Independent Variables:		
Innovation	−86.443^***^	117.701^*^
Advertising	−11.390^***^	−13.881
Personal Selling	8.217^***^	12.084
Comprehensive Sustainability	15.174^***^	26.082^***^
Interaction Items:		
Comprehensive Sustainability*Innovation		−536.494^***^
Comprehensive Sustainability*Advertising		5.540
Comprehensive Sustainability*Personal Selling		−8.087
Control Variables:		
Ln (Firm Age)	−5.702^***^	−5.530^***^
Ln (Asset Size)	1.197	1.260
Ln (Total Number of Employees)	−1.254	−1.557
Debt to Net Worth Ratio	−0.009^***^	−0.009^***^
Model-fitting metrics:		
*R* ^2^	0.2479	0.2664
Adjusted *R*^2^	0.2309	0.2435
*F* value	14.63***	11.62^***^
Observations	364	364

***p < 0.01, **p < 0.05, and *p < 0.10, two tailed test.

Model 6 is the regression of profitability on innovation, advertising, personal selling, comprehensive sustainability and four control variables, from which we can see that innovation and advertising’ s impact is negative and personal selling and sustainability’s impact is positive. Model 7 adds three additional interactive items of sustainability and innovation, advertising, and personal selling to Model 6. The result shows that only the regression coefficient of the interaction term between sustainability and innovation is negatively significant, while the regression coefficients of the other interaction items are not significant. Besides, for the four control variables, the significance of coefficients is also in accordance with original models. Consequently, after replacing the traditional sustainability score with the comprehensive sustainability score, the results are still consistent, which proves the robustness of this study.

#### 4.3.2. Changing the data source and parameter estimation method

In this part, we further test the robustness by changing the data source of sustainability variable and the estimation method. Sustainability is a key independent variable in this study, and the accuracy and objectivity of its measurement directly affect the research conclusion. In the study above, we used Wind data to measure sustainability. In China, Sino-Securities ESG Index is another highly recognized sustainability score data, which comprehensively considers the internal management system, business objectives, green products, external certification, violations and other factors, and the basic data used is mainly based on the public disclosure data of listed companies, and integrates auxiliary information such as social responsibility reports, sustainable development reports, national regulatory announcements, news media data, etc. We convert the original nine grades of C/CC/CCC/B/BB/BBB/A/AA/AAA into numerical values from 1 to 9, respectively, to simplify the quantitative analysis. To be specific, C has the lowest grade and is assigned the value of 1. AAA has the highest rating and is assigned the value of 9. Among the other grades, CC grade is assigned the value of 2, CCC grade is assigned the value of 3, B grade is assigned the value of 4, BB grade is assigned the value of 5, BBB grade is assigned a value of 6, A grade is assigned the value of 7, and AA grade is assigned the value of 8. We use the robust or sandwich estimator of variance method to run the regression models to further eliminate some types of misspecifications, which uses (sigma-hat_*j*)^2 = {*n*/(*n*-*k*)}(*u*_*j*)^2 as an estimate of the variance of the *j*th observation, where *u*_*j* is the calculated residual and *n*/(*n*-*k*) is included to improve the overall estimate’s small-sample properties, and the results are shown in Table 6.

**Table 6:** Robust test results by changing data source and estimation method.

Dependent variable: Profitability	Model 8	Model 9
Constant	58.078^***^	55.281^***^
Independent Variables:		
Innovation	−80.223^***^	−2.807
Advertising	−10.636^***^	−18.334^*^
Personal Selling	7.079^**^	19.916
Sino-Securities Sustainability	8.031^**^	14.017^***^
Interaction Items:		
Sino-Securities Sustainability*Innovation		−169.651^**^
Sino-Securities Sustainability*Advertising		13.446
Sino-Securities Sustainability*Personal Selling		−21.367
Control Variables:		
Ln (Firm Age)	−7.389^***^	−7.591^***^
Ln (Asset Size)	1.610	1.988
Ln (Total Number of Employees)	−0.922	−1.368
Debt to Net Worth Ratio	−0.010^**^	−0.008^***^
Model-fitting metrics:		
R^2^	0.2327	0.2447
F value	19.14***	82.31^***^
Observations	350	350

***p < 0.01, **p < 0.05, and *p < 0.10, two tailed test.

As with the models in the robustness test of 4.3.1, Model 8 is the regression of profitability on innovation, advertising, personal selling, Sino-Securities sustainability and four control variables, from which we can see that innovation and advertising’s impact is still negative and personal selling and sustainability’s impact is still positive. Model 9 adds three additional interactive items and the result again manifests that only the regression coefficient of the interaction item between sustainability and innovation is negatively significant, while the regression coefficients of the other interaction items are not significant. In addition, for the four control variables, the significance of coefficients is also in accordance with original models and the robustness test in Section 4.3.1. Therefore, we can draw the conclusion that when the Wind sustainability score is replaced by the Sino-Securities sustainability index, the results are still consistent, which once again proves the strong robustness of this research.

#### 4.3.3. Quantile regression results with bootstrap

In order to ensure the robustness of this study and observe the impact of innovation, advertising, personal selling, and sustainability on profitability at different profitability levels, we further analyzed the data using quantile regression and bootstrap methods. The number of bootstraps is set to 100, and the median regression results are shown in Table 7.

**Table 7:** Median regression results.

Profitability	Coef.	Std. Err	*t*	*p* > |*t*|	[95% Conf. Interval]	
Constant	61.421	12.956	4.74	0.000	35.941	86.901
Independent Variables:						
Innovation	−81.662	30.384	−2.69	0.008	−141.418	−21.906
Advertising	−7.117	2.308	−3.08	0.002	−11.657	−2.578
Personal Selling	6.494	2.144	3.03	0.003	2.278	10.710
Sustainability	9.898	3.305	2.99	0.003	3.397	16.398
Control Variables:						
Ln (Firm Age)	−5.167	1.459	−3.54	0.000	−8.036	−2.297
Ln (Asset Size)	−0.699	0.662	−1.06	0.292	−2.002	0.603
Ln (Total Number of Employees)	0.403	0.887	0.45	0.650	−1.342	2.148
Debt to Net Worth Ratio	−1.687	1.049	−1.61	0.109	−3.750	0.376
Model-fitting metrics:						
Pseudo *R*^2^	0.1472					
Observations	364					

**Figure 3:**
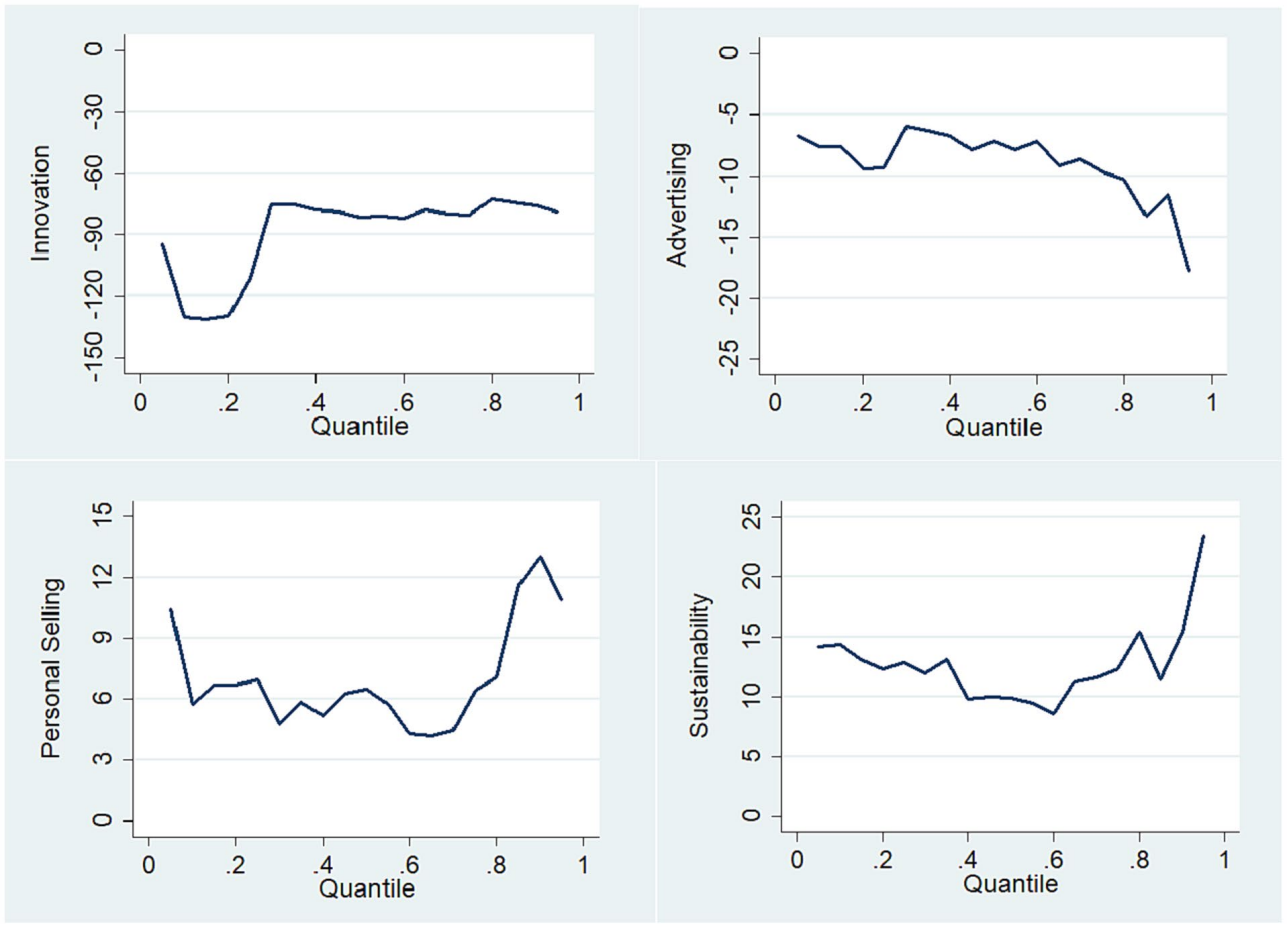
Quantile regression results.

As can be seen from Table 7, the impact of innovation and advertising on profitability remains significantly negative in terms of median, while the influence of personal selling and sustainability on profitability remains significantly positive. This is consistent with the results of the OLS regression above. We further demonstrate quantile regression results in Figure 3 of the impact of innovation, advertising, personal selling, and sustainability on profitability. At all quantile levels, the regression coefficients of innovation and advertising are both less than 0, while the regression coefficients of personal selling and sustainability are both greater than 0. This again shows that the results of this study have strong robustness.

## 5. Discussion

### 5.1. The resource allocation of innovation and marketing and firm profitability

The epidemic of the new crown pneumonia has had a serious impact on the world economy (73–75). However, the healthcare industry has certainly been a beneficiary industry amid this pandemic and brought with huge market demand. How to seize this opportunity and make the right innovation and marketing strategies is an important issue for enterprises.

The role of innovation in business performance and economic development has been widely recognized (14, 15, 76). However, from investing resources in research and development to bringing new technologies to market, it is often a long-term process, which determines that innovation should be a long-term behavior (77). On the other hand, as Link (18) and Kessler (19) have argued, market competition and the external environment are also important factors that should be considered in the innovation decisions of enterprises. When faced with a sudden huge market opportunity, if the competitors of a firm have obtained higher sales performance and market share through the adjustment of resource allocation, and this firm still insists to focus on R&D, it will inevitably be detrimental to its financial performance and competitiveness. Particularly, for the industries that have benefited greatly from major public health emergencies, the market demand has increased significantly, and companies are likely to be outperformed by competitors if they fail to seize the opportunity. At the level of firm practice, empirical data also confirm this negative relationship between innovation and profitability. For enterprises that benefit greatly from major public health emergencies, increasing their ability to respond to market dynamics and appropriately adjusting the investment of innovation resources according to the market environment will be more conducive to improving their current performance. However, in the long run, we still believe that companies should maintain and increase investment in innovation to improve their core competitiveness.

Compared to the internal orientation character of innovation, advertising and personal selling are directly market-oriented and have more advantages in responding to the market opportunities that suddenly arise in the market. Of the two, advertising of healthcare industry can not only enable consumers to fully understand product and service information and enhance health awareness, but also facilitate the communication and concordance between customers and doctors (78, 79). If the advertising strategy is well devised and deployed, it may foster provider-patient engagement initiatives and offer healthcare providers opportunities to dramatically improve their sales and performance by successfully engaging current and prospective patients and hastening exchange (80, 81). However, advertising in the healthcare industry is sometimes not objective and can be misleading, creating unrealistic expectations for consumers (82). Some healthcare industry consumers may feel that while commercial health advertising is helpful, it is also confusing, with many participants also holding mistaken beliefs around other elements of the commercial healthcare advertising (83). The chaos of advertising in the healthcare industry, as well as consumer misunderstanding of advertising for healthcare products, has dampened the positive effect of advertising on profitability. Besides, in order to deal with this problem, various measures have been taken by the regulatory agencies all over the world to effectively control and curb the misleading or false claims through strict regulations, which also increase the cost of advertising activities (84, 85). Moreover, in major public health emergencies, the demand for healthcare products is increased significantly, and consumers’ active product search behavior is also increased correspondingly, which may reduce the effectiveness of advertising to a certain extent. Ultimately, as shown in this paper, these factors make the output of advertising lower than the cost of input, and thus has a negative impact on profitability. For enterprises that have benefited greatly from major public health emergencies, paying attention to the correct promotion of advertising, and keeping a low profile, and appropriately reducing advertising expenditure will be more conducive to improving the efficiency and return on investment of resources.

Although the audience of advertising is very broad, it is relatively weak in terms of public relations with focused customers. To supplement this deficiency, personal selling is another important marketing tool often deployed in the healthcare industry (86). Healthcare products and services have a certain degree of professionalism, whose main target customers are hospitals, pharmacies, health care institutions, local governments, and other organizations with large procurement batches, which determines that personal selling would play an important role in the marketing mix (87–89). Especially in China, affected by the social and cultural factors represented by “GUANXI” (also called Personal Connections), if sales personnel could maintain good relationships with important customers such as local governments, hospitals, pharmacies, and health management companies, it will also have a positive role in promoting product sales (90–92). In fact, the sale skills of sales professionals in the healthcare industry and the management of GUANXI with customers are common in countries around the world, except some differences in ethical or legal norms (93, 94). Much of the literature in recent years also proves that the personal characteristics and abilities of pharmaceutical sales representatives have a significant impact on market share and sales performance (36, 37). Therefore, for companies benefiting from major public health emergencies, the personal selling targeted at focused customers would outperform large-scale advertising in improving profitability.

In a word, the market and consumer demand for healthcare products have increased significantly during major public health emergencies, and increasing resource investment and training in personal selling is more conducive to fully grasping market opportunities, thereby improving the profitability of enterprises. Consequently, it is not very unexpected that the impact of investing in personal selling on the profitability of enterprises in the current period is positive, while the investment in innovation and advertising would cause negative impacts.

### 5.2. The impact of sustainability and its moderating role

The major public health emergency is a kind of social disaster which would lead to serious losses of country, firms, and individuals. In this context, although some industries and companies may profit from the increase of market demand, the public still expects them to take the initiative to assume social responsibility rather than make lots of money during the disaster. In this context, enterprises increase sustainability investment and actively assume social responsibility are also a kind of marketing and publicity for themselves (95, 96). Especially when the industry is mixed with fishes and dragons and the public has doubts about the reputation of the industry, the investment of sustainability of healthcare companies will be conducive to the improvement of brand influence and business performance. From the statistical results of this study, the impact of sustainability on corporate profitability is twofold. On one hand, it can directly promote the improvement of corporate profitability. On the other hand, it also significantly moderates the relationship between innovation and profitability.

In hierarchical regression models, sustainability has a significant positive impact on corporate profitability, which verifies our analysis above. Specifically, among the three dimensions of sustainability, the impact of environmental sustainability on profitability is negative, while the impact of social sustainability and governance sustainability on profitability is positive. This suggests that the three dimensions of sustainability are not aligned in their directions. While the pursuit of sustainability can increase profitability, investment in environmental sustainability is still negative if viewed solely from the perspective of improving profitability. This result is also simple to understand: social sustainability and governance sustainability are oriented to the internal and external governance structure of the enterprise, and have a direct relationship with operation, products, market, and customers, so that it is easier to improve profitability by investing in these areas. But environmental sustainability is not the case. At least for now, the output of enterprises’ investment in environmental system and management system construction, resource conservation and protection, reduction of harmful emissions, green building and green finance is still lower than cost. How to make the investment of environmental sustainability to have a greater support for profitability remains one of the key issues for the healthcare industry and companies. In fact, regardless of whether a company’s investment in environmental sustainability results in a favorable return on investment, the image of social sustainability and governance sustainability is even more important for companies that profit a lot from a major public health emergency. Increased investment in social sustainability and governance sustainability is better for companies to make greater profits in a disaster environment.

We also find that sustainability may moderate the negative impact of innovation on profitability during the moderating effect analysis, which is somewhat unexpected while makes sense. Sustainability has been one of the key goals pursued by many companies since this century, and it emphasizes long-term development by conserving resources, protecting the environment, and establishing good internal and external relationships. The more sustainable companies are, the more emphasis is placed on long-term development through innovation, rather than reducing investment in innovation for reasons such as seizing current market opportunities and the short-term output efficiency of innovation. Ultimately, this leads to a more negative impact of innovation on current profitability in highly sustainable companies. Further analysis manifests that this moderating effect of sustainability is mainly caused by social sustainability, which primarily measures the performance of enterprises in terms of employment relations, occupational health, production safety, product quality, customer privacy protection, and community public welfare investment and reflects the responsibility and friendliness of enterprises at the social level. Companies with high social sustainability have a greater negative impact of their innovations on profitability than firms with low social sustainability as their innovation inputs are more long-term and less market-sensitive. In contrast, environmental sustainability and governance sustainability do not have a significant impact on the relationship between innovation and profitability, which means that the negative impact of corporate innovation investment on profitability will not change depending on environmental sustainability and governance sustainability. Furthermore, the level of sustainability also will not have a significant moderating effect on the profitability impact of advertising and personal selling.

In summary, for enterprises in the industries that profit a lot from major public health emergencies, it is beneficial for them to better seize market opportunities and improve business performance by appropriately tilting resource allocation towards sustainability. Businesses may consider shifting resources from innovation to social sustainability and governance sustainability to improve current profitability, which is strikingly different from the general context that emphasizes increasing profitability through innovation, marketing, and sustainability investments together.

## 6. Conclusion

The COVID-19 has affected many firm characteristics including firm performance, firm value, governance structure, dividend, liquidity, and leverage level (97, 98). Therefore, it is interesting to study how companies respond and the effects of these countermeasures. Although most industries will suffer losses during major public health emergencies, public health-related industries have ushered in lots of market opportunities. This article conducted research on companies in China’s healthcare industry during the coronavirus pandemic and found some interesting conclusions in the resource allocation strategies, which are not only consistent with but also supplementary to the previous researches on innovation, marketing, sustainability, and financial performance in general context. On one hand, we found that personal selling, social sustainability, and governance sustainability have a significant positive impact on profitability, just as the situation of many industries in the general context. On the other hand, the conclusion that environmental sustainability, innovation, and advertising have been shown to have a negative impact on profitability is worth pondering. For companies that benefit greatly from major public health emergencies, how to improve the resource efficiency of innovation and advertising tools, and balance environmental protection and financial performance remains an important issue to be addressed by the firms, industrial organizations, and government. Especially for the enterprises with high social sustainability, mechanisms should be put in place to help them reverse the negative impact of innovation on profitability. Finally, in general, for companies in industries that have benefited greatly from major public health emergencies, we believe that a shift in resource allocation from innovation, advertising, and environmental sustainability to personal selling, social sustainability, and governance sustainability will be more conducive to improving profitability. However, for public health regulatory authorities, it is necessary to strengthen the supervision of sales representatives of health care enterprises, hospitals, public health organizations, etc., and appropriately subsidize the innovation of enterprises to enhance their innovation motivation.

There are two main limitations in this study. First, in terms of sample selection, we used the data of listed companies of China’s healthcare industry, which does not include the small and medium-sized enterprises that unlisted on the stock market. Due to the limited sample size and scope, whether the result is suitable for other countries and industries also needs to be further verified. Moreover, like other empirical studies based on secondary data, our models may also suffer endogenous problems caused by the omission of potential explanatory variables and the correlation between residuals and independent variables. Second, this paper is a preliminary study about the industries that massively benefited from major public health emergencies and just focuses on the resource allocation strategy of innovation, marketing, and sustainability, while lacks of deep research in the specific mechanism and processes of innovation, advertising, and personal selling on profitability. We hope that in the future, more scholars and studies may pay attention to these relevant industries and corporate behavior in this special context and the research can focus on at least two directions. One is to study in-depth about the impact mechanism of these enterprises’ resource allocation and other competition strategies on performance in the context of major public health emergencies. The second is to discuss about how these industries and enterprises that have benefited massively from the major public health emergencies can achieve long-term sustainable development when the disaster ended.

## Data availability statement

Publicly available datasets were analyzed in this study. This data can be found here: https://www.wind.com.cn/en/.

## Author contributions

GX and CZ: conceptualization, project administration, and funding acquisition. GX: methodology and software. GX and YZ: validation. GX, CZ, QF, and XT: writing – original draft preparation. QF, XT, and YZ: writing – review and editing. QF and XT: supervision. All authors contributed to the article and approved the submitted version.
